# Malnutrition and Frailty Are Critical Determinants of 6-Month Outcome in Hospitalized Elderly Patients With Heart Failure Harboring Surgically Untreated Functional Mitral Regurgitation

**DOI:** 10.3389/fcvm.2021.764528

**Published:** 2021-12-02

**Authors:** Masakazu Miura, Shinichi Okuda, Kazuhiro Murata, Hitoshi Nagai, Takeshi Ueyama, Fumiaki Nakao, Mototsugu Shimokawa, Takeshi Yamamoto, Yasuhiro Ikeda

**Affiliations:** ^1^Department of Rehabilitation, Yamaguchi Prefectural Grand Medical Center, Hofu, Japan; ^2^Division of Nursing and Laboratory Science, Yamaguchi University Graduate School of Medicine, Ube, Japan; ^3^Ultrasonography Center, Yamaguchi Prefectural Grand Medical Center, Hofu, Japan; ^4^Department of Cardiology, Yamaguchi Prefectural Grand Medical Center, Hofu, Japan; ^5^Department of Biostatistics, Yamaguchi, University Graduate School of Medicine, Ube, Japan

**Keywords:** functional mitral regurgitation (FMR), heart failure, older people, body mass index, malnutrition, frailty

## Abstract

**Background:** Hospitalized patients with acute decompensated heart failure (ADHF) frequently exhibit aggravating mitral regurgitation (MR). Those patients do not always undergo surgical mitral valve repair, but particularly in the elderly, they are often treated by conservative medical therapy. This study was aimed to investigate factors affecting 6-month outcomes in hospitalized patients with heart failure (HF) harboring surgically untreated MR.

**Methods:** We screened the presence of MR in hospitalized patients with HF between September 2017 and May 2020 in the Yamaguchi Prefectural Grand Medical (YPGM) center. At the time of discharge of these patients, individuals with surgically unoperated MR, including primary and secondary origin, were consequently recruited to this single-center prospective cohort study. The patients with severe MR who undergo surgical mitral valve treatment were not included in this study. The primary endpoint was all-cause readmission or all-cause death and the secondary endpoint was HF-related endpoint at 6 months after discharge. The Cox proportional hazard regression analyses were employed to assess the predictors for the composite endpoint.

**Results:** Overall, 489 patients with ADHF were admitted to the YPGM center. Of those, 146 patients (30% of total patients with HF) (median age 83.5 years, 69 men) were identified as harboring grade II MR or greater. Consequently, all the recruited patients were diagnosed as functional MR. During a median follow-up of 186.0 days, a total of 55 patients (38%) reached the primary or secondary endpoints (HF death and readmission in 31 patients, other in 24 patients). As a result of multivariate analysis, geriatric nutritional risk index [hazard ratio (HR) = 0.932; 95% CI = 0.887–0.979, *p* = 0.005], age (HR = 1.058; 95% CI = 1.006–1.112, *p* = 0.027), and left ventricular ejection fraction (HR = 0.971; 95% CI = 0.945–0.997, *p* = 0.030) were independent predictors of all-cause death or all-cause admission. Body mass index (HR = 0.793; 95% CI = 0.614–0.890, *p* = 0.001) and ischemic heart disease etiology (HR = 2.732; 95% CI = 1.056–7.067, *p* = 0.038) were also independent predictors of the HF-related endpoints.

**Conclusion:** Malnutrition and underweight were substantial predictors of adverse outcomes in elderly patients with HF harboring surgically untreated moderate-to-severe functional MR.

## Introduction

Heart failure (HF) is becoming a common disease in our aging society. Hospitalized patients often exhibit significant mitral regurgitation (MR), an aggravating factor of HF (1). Although the severity of MR is known to be associated with poor prognosis, surgical repair of MR is not always chosen in clinical settings. In this case, guideline-based recommendations do not decide this choice, but patient–individual-related factors, including comorbidities, physical, and social activity, restrict directions for treatment.

When mitral valve degeneration is the primary cause of MR, surgical mitral valve repair is the most curative treatment, if cardiac contractility is preserved (2). However, secondary MR, also called functional MR (FMR), is more common in the acute exacerbation of HF. In this regard, recent advances in transcatheter edge-to-edge repair (TEER) technology (3) have been drawing attention to treating FMR associated with left ventricular dysfunction and remodeling. Because of its less invasiveness, TEER may apply to elderly patients with HF complicated by moderate-to-severe MR. However, it is also reported that the therapeutic effect of TEER is limited without adequate standard pharmacotherapy (4). Currently, dissemination of the procedure is not yet sufficient.

In addition, elderly patients with HF often were categorized as HF with preserved ejection fraction (HFpEF) (5, 6). In this regard, atrial hamstringing FMR associated with left atrial enlargement has also come to the fore as a cause of FMR associated with HFpEF (2). There are currently few data available for a recommendation of surgical repair in patients with atrial hamstringing FMR. Furthermore, older patients with HF harboring MR often have multiple comorbidities, physical frailty, and cognitive impairment that increase the risk of surgical intervention (7–10). Therefore, currently, conservative medical therapy often becomes the only remaining choice.

In the clinical settings, once the cardiac overload on admission has been removed by pharmacotherapy, patients with HF harboring moderate-to-severe MR become a less symptomatic chronic state at the time of discharge. We believe that it is essential to help such patients avoiding symptomatic deterioration in daily life. In this regard, the heart team must make a holistic decision to predict the optimal patient outcome and reflect an outpatient care during the follow-up period. Assessing physical and nutritional status for cardiac rehabilitation (CR) is also essential for the multidisciplinary treatment of HF (11).

In this study, we investigate the predictive factors in hospitalized patients with HF harboring moderate-to-severe MR.

## Methods

### Study Population

From September 1, 2017 to May 31, 2020 at the Yamaguchi Prefectural Grand Medical (YPGM) center, patients who were admitted to the emergency room due to acute decompensated HF (ADHF) and have grade II MR or greater and received CR were employed in this cohort study. For entry of this study, at least two expert cardiologists reviewed echocardiography. They decided whether the patient was eligible for the investigation by assessing that HF aggravation was associated with exacerbation of MR. Indication for surgical repair of MR was discussed in the heart team conference and patients eligible for surgical repair underwent mitral repairment (*n* = 4). Eligibility for surgery was determined by at least five expert cardiology physicians and two expert cardiac surgeons. Patients who did not choose surgical repair despite the expert opinion or were diagnosed as ineligible for surgical repair were included in this cohort study according to the Japanese Circulation Society/Japanese Heart Failure Society guidelines (12). The following patients were excluded from this entry due to the complicated nature of disease pathophysiology. Exclusion criteria were: (i) patients who undergo maintenance dialysis due to end-stage renal failure, (ii) patients classified as clinical scenario 4 or 5 (13), or (iii) patients who lost follow-up.

This study was performed in accordance with the Declaration of Helsinki and approved by the local institutional board at the YPGM center (ID: 2017–2019). All the patients received a written informed consent before registration.

### Echocardiography Study

Transthoracic echocardiography was performed on admission and 2 weeks after entry in a stable condition. First, the nature of MR was classified as primary or secondary MR and presence or absence of ischemic heart disease (IHD). Second, the severity of MR was divided into five levels by semiquantitative assessment, i.e., grade 0: none to trace MR, grade 1: mild MR, grade 2: moderate MR, grade 3: moderate-to-severe MR, and grade 4: severe MR. Quantitative assessments of MR severity were obtained by evaluating the effective regurgitant orifice area (EROA), the tethering height, and the vena contracta of the MR. Left ventricular ejection fraction (LVEF), estimated by Simpson's biplane formula, left ventricular end-diastolic dimension (LVDd), left ventricular end-systolic dimension (LVDs), left atrial dimension (LAD), E/A ratio, E/e' ratio, tricuspid annular plane systolic excursion (TAPSE), transtricuspid pressure gradient (TRPG), and left atrial volume index (LAVI) were also obtained. Patients were categorized into three groups by LVEF, namely, HF with reduced LVEF (HFrEF) (LVEF < 40%), HF with mid-range LVEF (HFmrEF) (LVEF 40–49%), and HFpEF (LVEF ≥ 50%) (14).

### Clinical Data Collection

The clinical characteristics of the patients were collected from medical records including age, sex, body mass index (BMI), the New York Heart Association (NYHA) on admission, length of hospital stay (days), prior hospitalization, living alone, use of nursing care insurance, cognitive function assessed by the Montreal Cognitive Assessment-Japanese version (MoCA-J) (15), past histories of orthopedic disease, stroke, chronic kidney disease, diabetes, and atrial fibrillation. The biochemical laboratory data were also obtained from medical records including brain natriuretic peptide (BNP) on admission, serum albumin (Alb), serum hemoglobin, serum creatinine, and C-reactive protein (CRP). The geriatric nutritional risk index (GNRI) was calculated by the formula of [1.489 × Alb (g/l) + 41.7 × body weight (kg)/ideal body weight (kg)] (16). The estimated glomerular filtration rate (eGFR) at discharge was calculated from the above variables. Details of pharmacotherapy of HF were confirmed at the time of discharge including angiotensin-converting enzyme inhibitor (ACEI)/angiotensin II receptor blocker (ARB), β-blocker, tolvaptan, loop diuretics, and mineralocorticoid receptor antagonists (MRAs). Systolic blood pressure (SBP) and mean blood pressure (MBP) were measured at discharge.

### Physical Function

The effectiveness of CR was evaluated 5 days before the discharge. These include the short physical performance battery (SPPB) test (17), a handgrip test by a grip strength meter (T.K.K.5401 GRIP-D; Takei, Tokyo, Japan), and the quadriceps isometric strength (QIS) test by a handheld dynamometer (MT-100 mobile; Sakai Med, Tokyo, Japan) (18). Exercise tolerance was assessed by the 6-minute walk test (6MWT) (19). The activity of daily living was assessed by calculating the Barthel Index (BI) (20). Frailty was assessed by the Kihon Checklist (KCL), defined by the Ministry of Health, Labor, and Welfare, Japan (21–23). The KCL consists of 25 questionaries; a higher KCL score indicates a higher risk of frailty, those with the range from 0 to 3 points as the non-frailty group, those with the range from 4 to 7 points as a prefrailty group, and those scores of ≥8 points were defined as the frailty group. Sarcopenia was also assessed by the diagnostic criteria of the Asia Working Group for Sarcopenia (24). Weaker grip strength (<26 kg for men, <18 kg for women), slower gait speed (<0.8 m/s), and lower skeletal muscle mass index (SMI) measured by bioimpedance analysis <7.0 kg/m^2^ for men and <5.7 kg/m^2^ for women were regarded as sarcopenia. Bioimpedance analysis was performed using a bioelectrical impedance analyzer (Inbody S10; Inbody Japan, Tokyo, Japan).

Bioimpedance analysis was not applicable for patients implanted with implantable cardioverter defibrillator (ICD) or pacemaker (26/146 patients, 18%). Moreover, the 6MWT was inappropriate for patients who could not walk more than 100 m (20/146 patients, 14%).

### Follow-Up and Endpoint for the Analysis

The primary endpoint was defined as the composite endpoints consisting of all-cause death or all-cause admission and the secondary endpoint was defined as HF death or HF admission. Six months after discharge from the hospital, patient status was surveyed by postcard to determine whether they had experienced any events.

### Statistical Analysis

Statistical analysis was performed by using the EZR on R commander (version 1.37) (25). Categorical baseline variables were expressed as number and percentage or continuous variables were expressed as mean ± SD or median [interquartile range (IQR): 25th to 75th percentiles]. The primary endpoints were all-cause mortality and all-cause readmission and secondary endpoints were HF-related death and HF-related readmission. The severity of MR significantly contributed to prognosis of the patient that was tested by the log-rank test and compared by the Kaplan–Meier curve before the multivariate Cox proportional hazard regression analysis.

Next, predictors of survival and readmission were identified by the univariate and multivariate Cox proportional hazard regression analyses. Independent variables for multiple modeling were selected from predictive factors with *p* < 0.20 using the univariate analysis and previously reported predictive factors (1, 26–29). A stepwise variable reduction method was used for the multivariate Cox proportional hazard regression modeling.

Results were summarized as hazard ratio (HR), 95% CI, and *p*-value. When the predictors of continuous variables were identified by the multivariate analysis, a receiver operating characteristic (ROC) analysis was employed to determine the optimal cut-off value acting as independent predictive factors, followed by the sensitivity, the specificity, and the area under the curve (AUC). Event-free ratios were estimated by the Kaplan–Meier method and compared by the log-rank test. A *p* < 0.05 was considered as statistically significant.

## Results

### Baseline Clinical Characteristics

**Figure 1:**
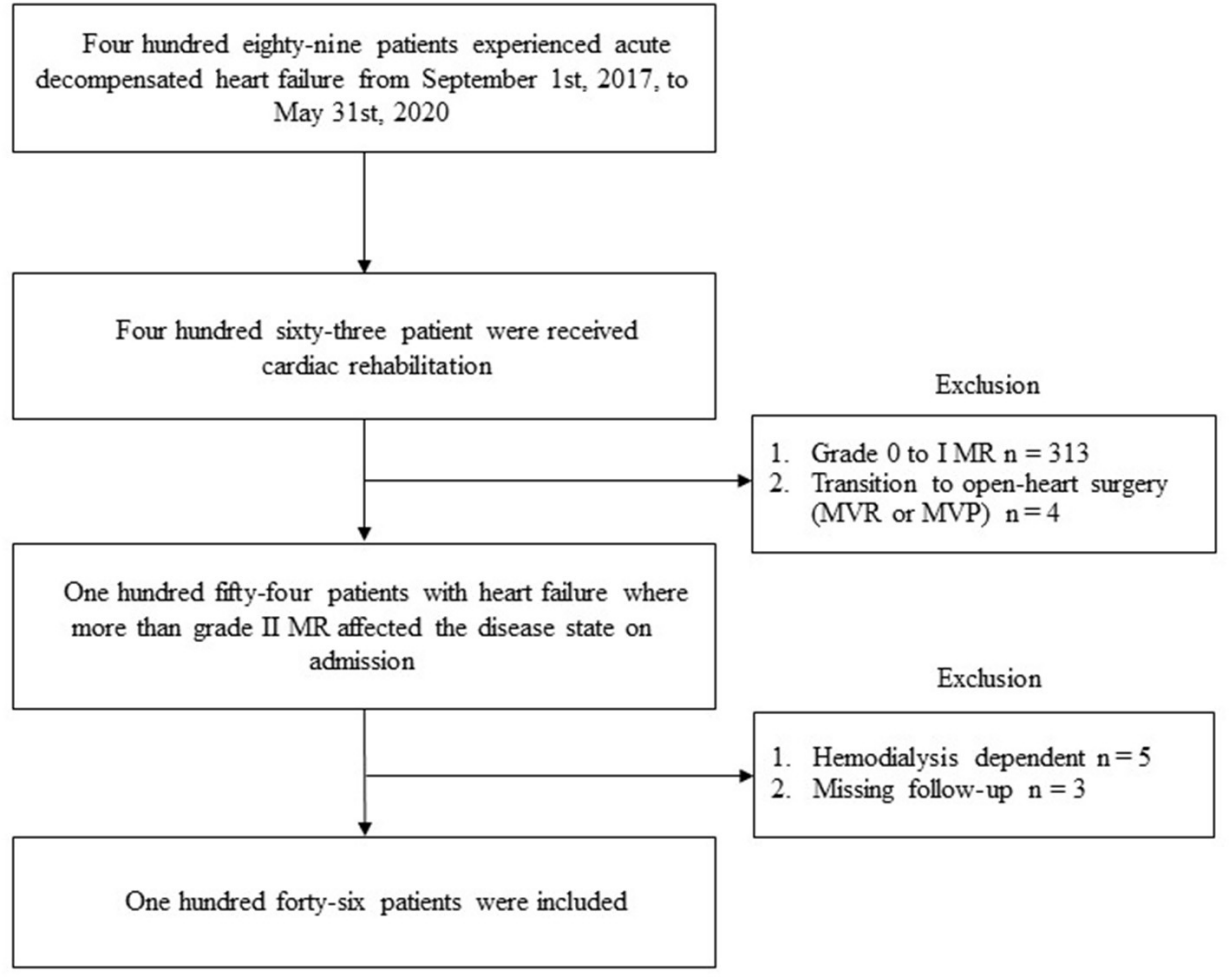
The flowchart of the study in patients with heart failure harboring moderate-to-severe MR in the Yamaguchi Prefectural Grand Medical center. MR, mitral regurgitation; MVR, mitral valve replacement; MVP, mitral valvuloplasty.

Overall, 489 patients with ADHF were admitted to the YPGM center. Of those, 146 patients (Figure 1) (30% of total patients with HF) (median age 83.5 years, 69 men) were identified as individuals harboring grade II MR or greater and enrolled in this study. Baseline clinical characteristics are shown in Table 1. All the patients expressed exacerbating symptoms of HF regarded as NYHA III/IV on admission. One in four patients lived alone and 40% of patients had been receiving nursing care insurance. The average length of hospitalization was 20 days. A total of 114 patients could return home at discharge and the rest had to be transferred to other CR hospitals due to insufficient physical and social activity recovery.

**Table 1:** Baseline clinical characteristics of 146 patients with ADHF harboring FMR.

	**Value**
**Demographics**	
Age, years	83.5 (72.3–88.0)
Male sex	69 (47)
BMI, kg/m^2^	20.2 (17.9–22.7)
NYHA class III/IV (on admission)	47/99 (32/68)
Living alone	35 (24)
Nursing care insurance	59 (40)
Length of hospital stay, day	20.0 (15.0–27.0)
Follow-up period, day	180.0 (109.8–180.0)
Prior hospitalization	72 (49)
Return to home	114 (78)
SBP, mmHg (at discharge)	114.4 ± 18.3
MBP, mmHg (at discharge)	83.6 ± 12.0
**Cognitive function**	
MoCA-J, points	18.5 (14.3–24.0)
**Co-morbidities**	
Orthopedic disease	51 (35)
Stroke	23 (16)
Hypertension	80 (55)
CKD	32 (22)
DM	42 (29)
Atrial fibrillation	61 (42)
IHD	47 (32)
**Transthoracic echocardiography (two weeks after admission)**	
**MR**	
Zero	11 (8)
I	26 (18)
II	67 (46)
III	40 (27)
IV	2 (1)
MR grade (continuous variable)	2 (1–3)
LVEF, %	45.0 (32.0–60.5)
HFrEF	65 (45)
HFpEF	58 (40)
HFmrEF	19 (13)
LVDd, mm	52.0 ± 8.8
LVDs, mm	38.5 (30.0–47.3)
LAD, mm	45.0 (40.0–50.0)
LAVI	58.0 (45.5–81.0)
E/e' ratio	16.0 (13.0–22.0)
E/A ratio	0.99 (0.65–1.46)
TRPG, mmHg	28.0 (23.1–35.0)
TAPSE, mm	16.1 ± 4.2
Tethering height, mm	9.0 ± 3.0
Vena contracta, mm	3.8 (3.0–5.0)
EROA, cm^2^	0.12 (0.07–0.18)
**Laboratory data**	
Serum albumin, g/dl	3.4 ± 0.5
Serum creatinine, mg/dl	1.03 (0.82–1.29)
eGFR, mL/min/1.73 m^2^	48.0 (36.0–63.0)
Serum hemoglobin, g/dl	11.5 (10.1–13.2)
BNP, pg/ml (on admission)	574.0 (299.5–967.3)
**Nutritional status (at discharge)**	
Geriatric nutrition risk index, points	89.2 ± 10.6
**Medication (at discharge)**	
ACE-I /ARB	102 (70)
Loop diuretics	111 (76)
β-blocker	102 (70)
MRAs	50 (34)
Tolvaptan	46 (32)
**Physical function (at discharge)**	
SPPB, points	8 (5–10)
6MWT, m	279.0 (195.5–350.0)
QIS, Nm/kg	0.66 (0.50–0.78)
Handgrip strength, kg	13.3 (8.5–19.8)
Sarcopenia	73 (50)
KCL, points	11.0 (7.0–14.0)
BI, points	85.0 (70.0–90.0)

*Values were shown as mean ± SD, median [interquartile range (IQR): 25th to 75th percentiles], n (%)*.

*ADHF, acute decompensated heart failure; FMR, functional mitral regurgitation; BMI, body mass index; NYHA, New York Heart Association; SBP, systolic blood pressure; MBP, mean blood pressure; MoCA-J, Montreal Cognitive Assessment-Japanese version; CKD, chronic kidney disease; DM, diabetes mellitus; IHD, ischemic heart disease; LVEF, left ventricular ejection fraction; HFrEF, heart failure with reduced ejection fraction; HFpEF, heart failure with preserved ejection fraction; HFmrEF, heart failure with mid-range ejection fraction; LVDd, left ventricular end-diastolic dimension; LVDs, left ventricular end-systolic dimension; LAD, left atrial dimension; LAVI, left atrial volume index; TRPG, transtricuspid pressure gradient; TAPSE, tricuspid annular plane systolic excursion; EROA, effective regurgitant orifice area; eGFR, estimated glomerular filtration rate; BNP, brain natriuretic peptide; GNRI, geriatric nutritional risk index; ACEI/ARB, angiotensin-converting enzyme inhibitor/angiotensin II receptor blocker; MRAs, mineralocorticoid receptor antagonists; SPPB, short physical performance battery; 6MWT: six minute walking test; QIS, quadriceps isometric strength; KCL, Kihon Checklist (see Supplementary File 1); and BI, Barthel Index*.

**Figure 2:**
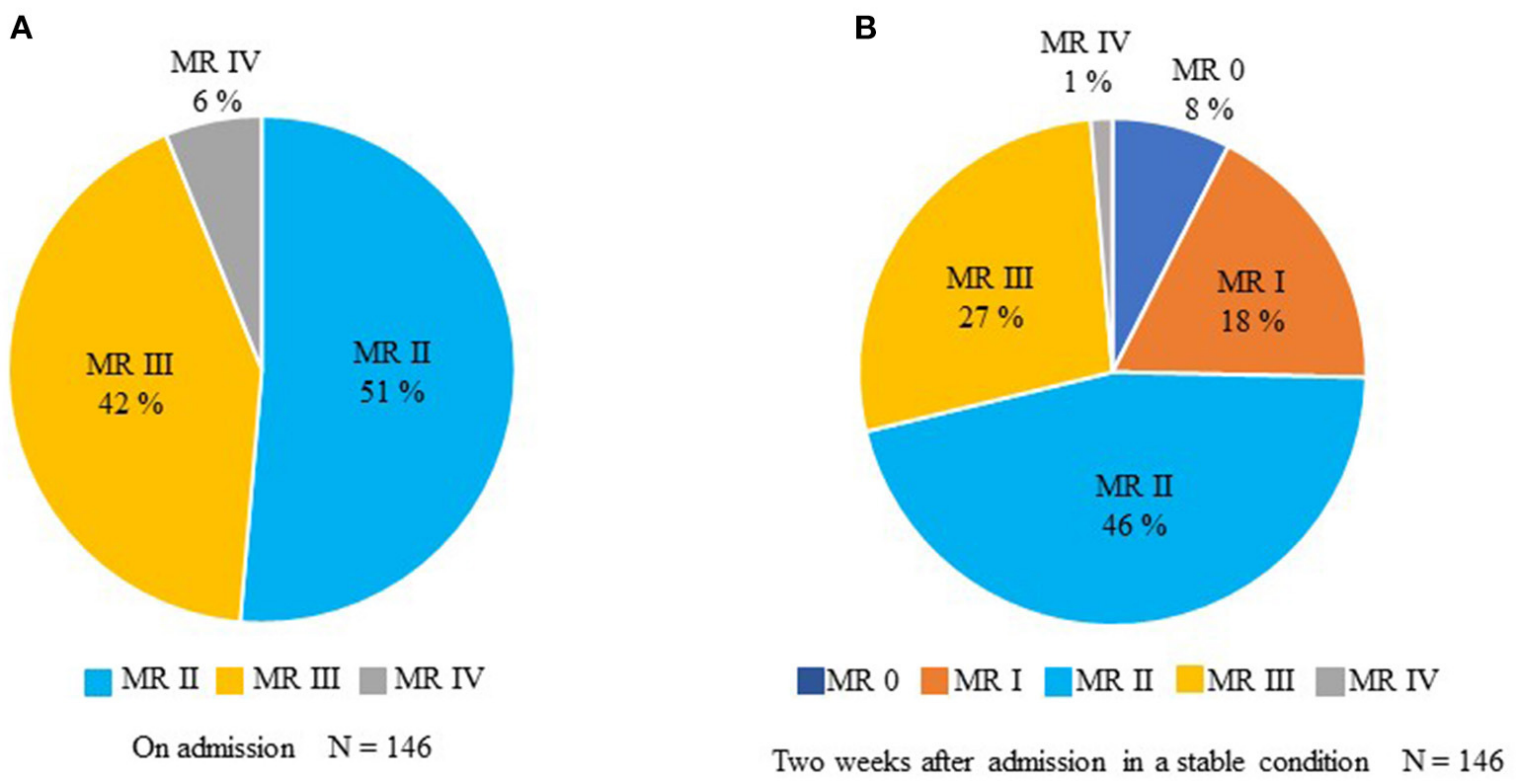
The temporal change in MR from admission to 2 weeks after entry in a stable condition. **(A)** Assessment of MR on admission. **(B)** Evaluation of MR 2 weeks after entry.

None of the patients with primary MR was included and all the recruited patients were diagnosed as secondary MR, i.e., FMR. Figure 2 shows the change of MR severity from admission to 2 weeks after admission; 108 (74%) patients have remained more than grade II MR. The rest of 38 patients (26%) of included patients exhibited clinically insignificant MR grades 2 weeks after admission. Concerning the left ventricular function, 45% of patients with HF revealed reduced EF (<40%), 13% of patients with HF showed mid-range EF (40–49%), and 40% of patients with HF exhibited preserved EF (≥50%).

**Figure 3:**
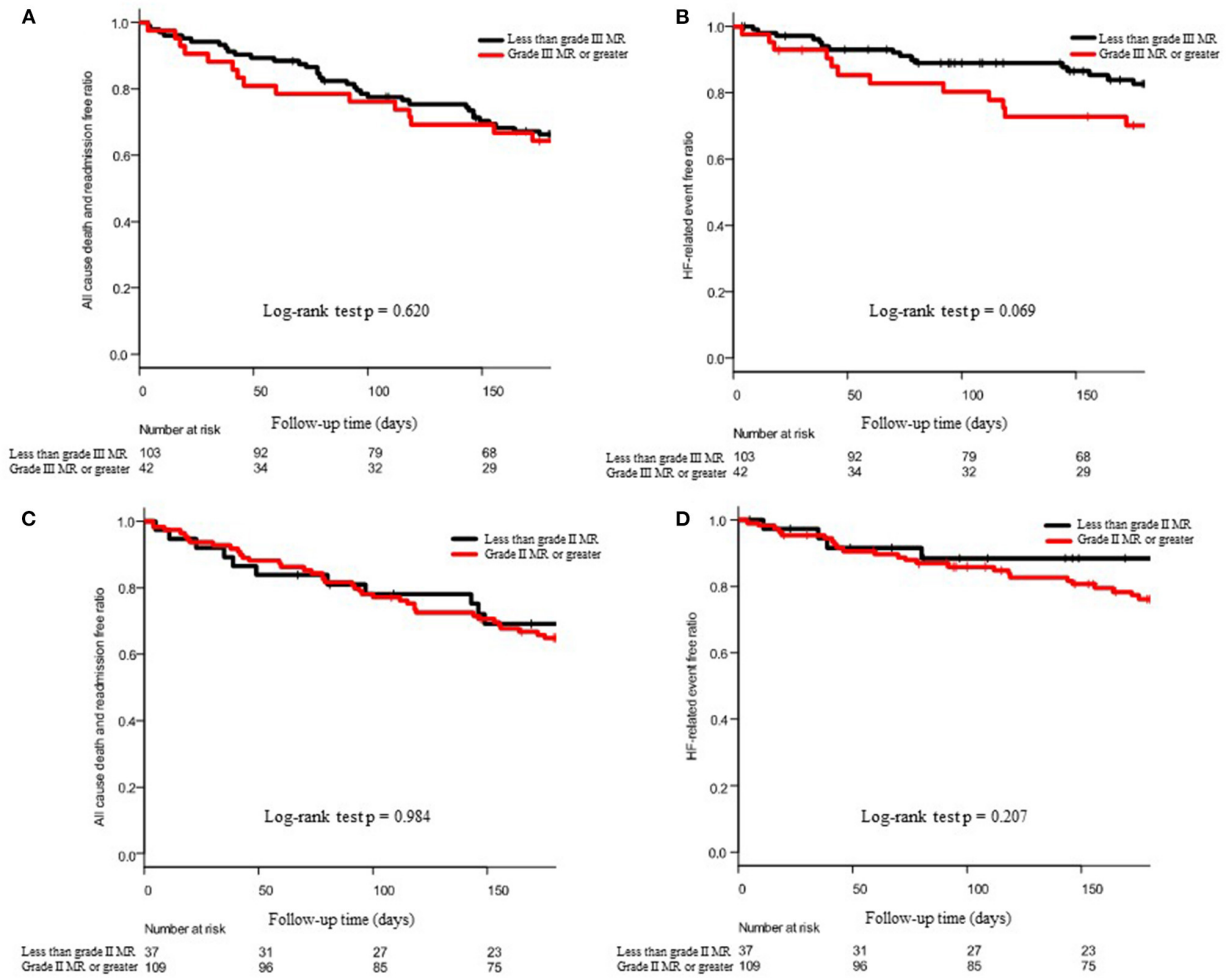
Effect of the severity of MR on 6-month outcomes. **(A,C)** show the Kaplan–Meier curve of all-cause death and admission and **(B,D)** show the Kaplan–Meier curve of HF-related death and admission. In **(A,B)**, patient groups were divided into the grade III MR or greater and less than grade III, and in **(C,D)**, patients were divided into the grade II MR or greater and less than grade II. The *p*-values of the log-rank test were described at the bottom of each panel.

Figures 3A,B show the 6-month outcomes of patients with MR of grade III or greater and those with MR of less than grade III. Figures 3C,D show the 6-month outcomes of MR patients with grade II or greater and MR patients with less than grade II. In both the cases, severity of MR was not a statistically significant factor.

The mean GNRI score was 89.2 ± 10.6, indicating that most patients had an intermediate or higher risk of protein–energy malnutrition. The median (IQR) MoCA-J was 18.5 (14.3–24.0), indicating that all the patients had mild-to-moderate cognitive impairment. Moreover, most patients had multiple comorbidities including orthopedic disease, stroke, hypertension, chronic kidney disease, diabetes, and atrial fibrillation. A total of 47 (32%) patients had IHD.

All the patients were treated by guideline-based standard pharmacotherapy except for angiotensin receptor-neprilysin inhibitor (ARNI) and sodium-glucose cotransporter 2 (SGLT2) inhibitor. These drugs had not yet been approved as HF treatment at the time of analysis in Japan. The mean SPPB was 8 (5–10), indicating that most patients had lower levels of lower limb muscle strength accompanying prefrail or frail physical activity. The higher KCL score and the lower BI score also support that most patients with HF harboring FMR had significant frailty and limited daily living activities.

### Overall Outcome

A total of 12 patients died during a median follow-up of 186.0 days (109.8–186.0) (HF in 8 patients, sepsis in 1 patient, acute peritonitis in 1 patient, multiple organ failure in 1 patient, and aspiration pneumonia in 1 patient). A total of 43 patients were readmitted to the hospital (worsening HF in 23 patients, aortic dissection in 1 patient, cerebral thromboembolism in 1 patient, pneumonia in 2 patient, orthopedic disease in 5 patients, cancer in 1 patient, and others in 10 patients); consequently, 31 patients (21%) reached HF-related endpoints. Although two patients with HF were harboring severe aortic stenosis (AS) in addition to FMR in this study, the presence of AS did not affect the primary and secondary endpoints.

### Predictive Factors of Primary and Secondary Composite Endpoints

Tables 2, 3 show the univariate and multivariate Cox proportional hazard regression analyses for the primary and secondary endpoints, respectively.

**Table 2:** The univariate and multivariate Cox proportional hazard regression analyses to predict composite endpoint after discharge of patients with ADHF harboring FMR.

**Variables**	**Univariate**			**Multivariate**		
	**HR**	**95%CI**	***p*-value**	**HR**	**95%CI**	***p*-value**
Age	1.489	0.862–2.572	0.154	1.058	1.006–1.112	0.027
Male sex	1.149	0.670–1.969	0.614			
BMI	0.430	0.243–0.760	0.004			
SBP	0.990	0.976–1.005	0.206			
IHD	1.524	0.892–2.603	0.123			
**Grade 0 MR (reference)**						
Grade III MR or greater	1.117	0.633–1.972	0.702			
LVEF	0.582	0.335–1.010	0.054	0.971	0.945–0.997	0.030
EROA	2.131	1.121–4.051	0.021			
BNP	1.881	1.075–3.291	0.027			
GNRI	0.511	0.292–0.895	0.019	0.932	0.887–0.979	0.005
Tolvaptan	2.334	1.359–4.007	0.002			
SPPB	0.740	0.424–1.291	0.289			

*The multivariate Cox proportional hazard regression analysis results were shown using a stepwise variable reduction method described in the Method section. The remaining univariate analysis variables, the p-value larger than 0.20, were listed in Supplementary File 2.*

*HR, hazard ratio; ADHF, acute decompensated heart failure; FMR, functional mitral regurgitation; BMI, body mass index; SBP, systolic blood pressure; IHD, ischemic heart disease; LVEF, left ventricular ejection fraction; EROA, effective regurgitant orifice area; BNP, brain natriuretic peptide; GNRI, geriatric nutritional risk index; SPPB, short physical performance battery*.

**Table 3:** The univariate and multivariate Cox proportional hazard regression analyses to predict HF-related endpoint after discharge of patients with ADHF harboring FMR.

**Variables**	**Univariate**			**Multivariate**		
	**HR**	**95%CI**	***p*-value**	**HR**	**95%CI**	***p*-value**
Age	1.590	0.765–3.304	0.214			
Male sex	0.784	0.378–1.628	0.514			
BMI	0.487	0.231–1.027	0.059	0.793	0.614–0.890	0.001
SBP	1.001	0.982–1.021	0.893			
Readmission	2.261	1.058–4.832	0.035			
IHD	1.797	0.897–3.599	0.098	2.732	1.056–7.067	0.038
**Grade 0 MR (reference)**						
Grade III MR or greater	1.748	0.869–3.517	0.117			
LVEF	0.600	0.289–1.248	0.172			
EROA	1.275	0.532–3.058	0.586			
BNP	2.336	1.093–4.994	0.029			
GNRI	0.432	0.201–0.926	0.031			
Tolvaptan	2.965	1.445–6.083	0.003			
KCL	2.647	1.238–5.658	0.012			
SPPB	0.515	0.236–1.125	0.096			

*The multivariate Cox proportional hazard regression analysis results were shown using a stepwise variable reduction method described in the Method section. The remaining univariate analysis variables, the p-value larger than 0.20, were listed in Supplementary File 3*.

*HR, hazard ratio; ADHF, acute decompensated heart failure; FMR, functional mitral regurgitation; BMI, body mass index; SBP, systolic blood pressure; IHD, ischemic heart disease; LVEF, left ventricular ejection fraction; EROA, effective regurgitant orifice area; BNP, brain natriuretic peptide; GNRI, geriatric nutritional risk index; KCL, Kihon Checklist; SPPB, short physical performance battery*.

**Figure 4:**
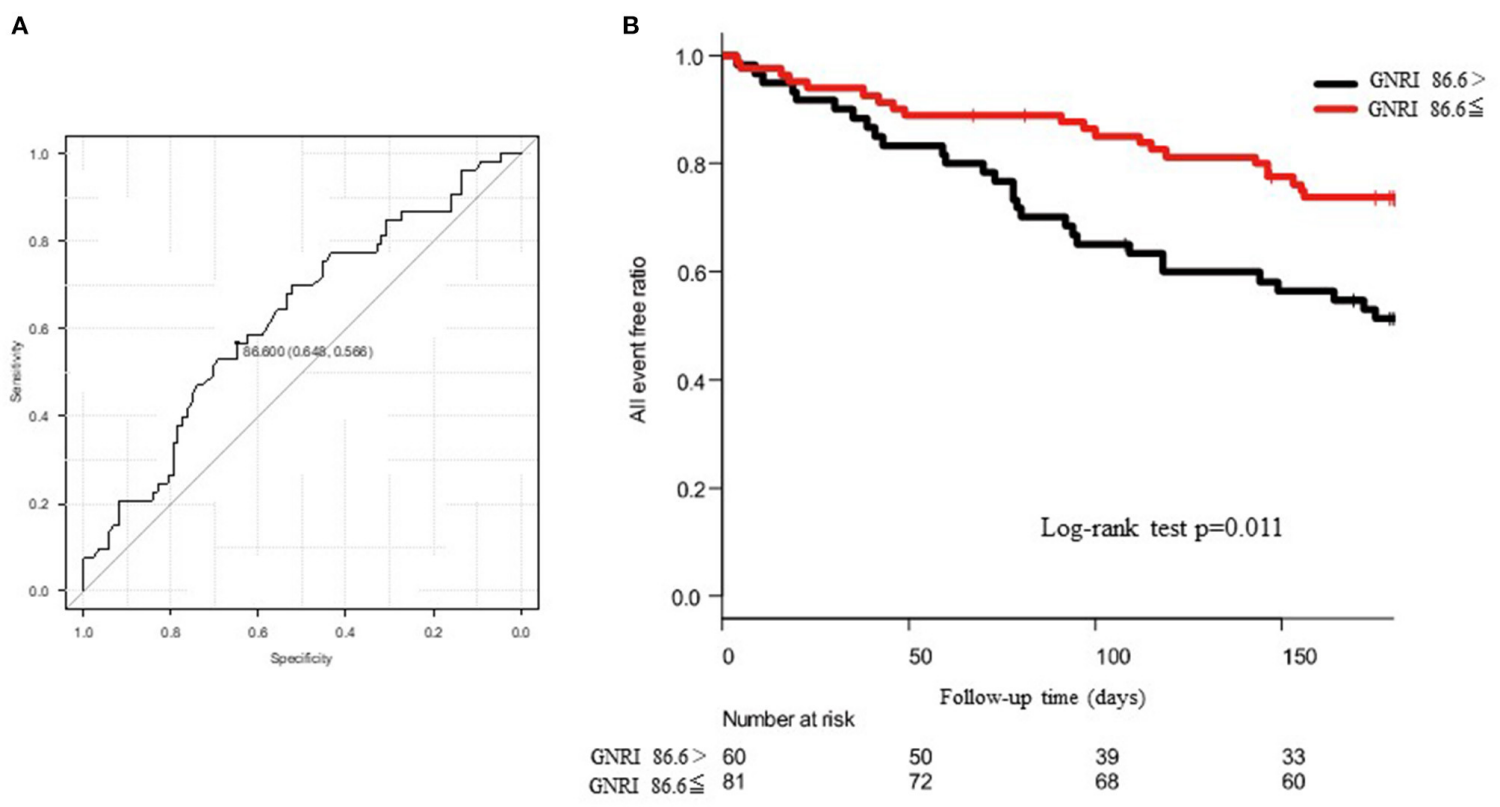
**(A)** shows the receiver operating characteristic (ROC) curve to predict the cut-off value of composite endpoints using the geriatric nutritional risk index (GNRI). The area under the curve (AUC) was 0.625 and 95% CI was from 0.530 to 0.721. When the cut-off value of GNRI was set to 86.6, the sensitivity and the specificity became 65 and 57%, respectively. **(B)** shows the Kaplan–Meier curve to evaluate the cumulative incidences of composite endpoints. The log-rank test revealed a *p*-value of 0.011.

For the all-cause death and admissions, BMI, EROA, BNP, GNRI, and use of tolvaptan were significant determinants of the all-cause death and admissions in the univariate analysis, as shown in Table 2. The multivariate Cox proportional hazard regression analysis revealed that GNRI, age, and LVEF were independent determinants of the all-cause death and admissions, as shown in Table 2. In the ROC analysis, when the cut-off value of the GNRI was set to 86.6, the sensitivity, specificity, and the AUC were 65, 57%, and 0.625 (95% CI = 0.530–0.721), respectively (Figure 4A). The Kaplan–Meier curve revealed a significantly higher incidence of the primary endpoints in patients with the GNRI <86.6 than in GNRI ≥ 86.6 (Figure 4B).

**Figure 5:**
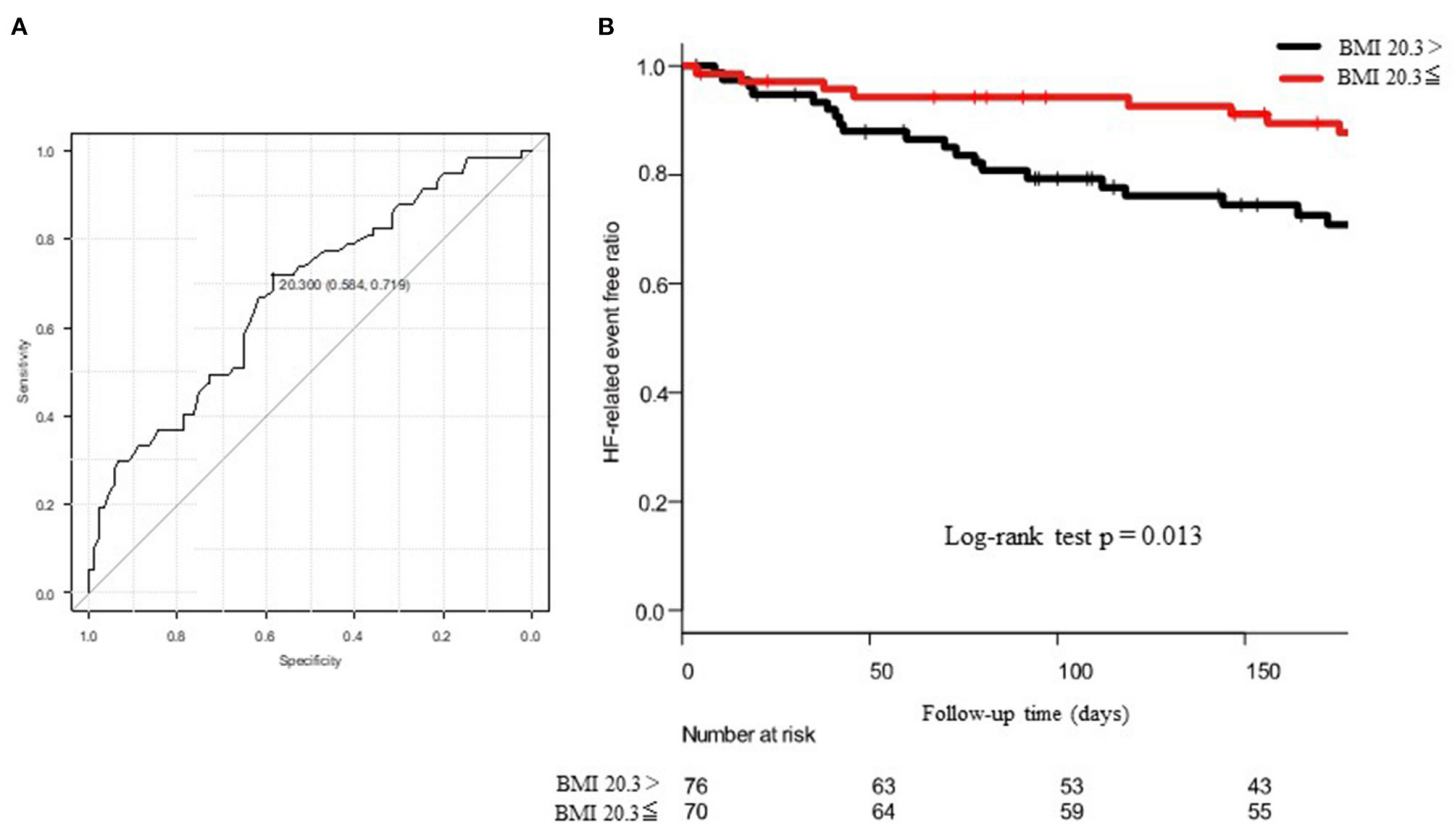
**(A)** shows the ROC curve to predict the cut-off value of HF-related endpoints using the body mass index (BMI). The AUC was 0.675 and 95% CI was from 0.586 to 0.765. When the cut-off value of BMI was set to 20.3, the sensitivity and the specificity became 58 and 72%, respectively. **(B)** shows the Kaplan–Meier curve to evaluate the cumulative incidences of HF-related endpoints. The log-rank test revealed a *p*-value of 0.013.

For the HF-related endpoints, readmission, BNP, GNRI, use of tolvaptan, and the KCL score were significant determinants of the HF-related endpoints in the univariate analysis. The multivariate Cox proportional hazard regression analysis revealed that etiology of BMI and IHD were independent determinants of the HF-related endpoints. In the ROC analysis, when the cut-off value of BMI was set to 20.3, the sensitivity, specificity, and the AUC were 58, 72%, and 0.675 (95% CI = 0.586–0.765), respectively (Figure 5A). The Kaplan–Meier curve revealed a significantly higher incidence of HF-related endpoint in patients with BMI <20.3 kg/m^2^ than those with BMI ≥ 20.3 kg/m^2^ (Figure 5B).

## Discussion

In this prospective observational cohort study of patients with ADHF harboring moderate-to-severe FMR, we found that a lower nutrition index and underweight were substantial predictors of 6-month all-cause mortality and HF-related composite outcomes. In addition, higher age and lower LVEF were associated with a worse outcome of all-cause death and admission and etiology of IHD was also associated with a worse outcome of HF-related death and admissions. Interestingly, severity of MR was not significantly associated with the primary and secondary outcomes. To the best of our knowledge, this is the first report describing that nutritional status is more important than the severity of MR *per se* in elderly patients with ADHF harboring moderate-to-severe FMR.

We initially intended to include all the moderate-to-severe etiologies of MR in elderly patients ADHF in this study. Consequently, all the patients were categorized as secondary MR at enrollment, underscoring that FMR plays a crucial role in the onset of ADHF. Of the 146 patients with FMR, 109 (74%) of patients with HF had more than grade II MR 2 weeks after admission, whereas 26% of patients with HF showed a transition to less than grade II MR 2 weeks after admission. These are typical characteristics of secondary MR in ADHF, as previously reported (26). Indeed, these secondary MR contains two categories of etiology, approximately half of the patients showed ventricular FMR with reduced EF (26, 29, 30) and the remaining half of the patients revealed atrial hamstringing FMR associated with atrial fibrillation and preserved or mid-range ejection fraction (26, 31–34). A higher incidence of atrial fibrillation in the latter category is consistent with such etiological origin.

It is noteworthy that severity of MR was not associated with primary and secondary outcomes in this study. This result is different from previous studies that reported that mild-to-severe secondary MR affected all-cause mortality and HF readmission (35). It is plausible that a relatively small number of analyzed patients, use of older-aged groups, short follow-up period, and predominance of female patients (53% of the patients) in this study may be associated with the difference from the previous studies. Indeed, the mean age of this study was more than 10 years older than those of earlier reports (35–37). The percentage of female patients was more prominent than those in previous reports (35–37). It has been reported that women have a 26% higher relative risk than men to be frail and have lower body weight in HF (38). Therefore, the characteristics of female patients with HF may override MR-related outcomes. In addition, in the previous studies, nutritional variables and physical and social parameters associated with CR were not used in the univariate and multivariate Cox proportional hazard regression analyses to predict composite endpoints after discharge of patients with ADHF. Therefore, it is plausible that the difference of used statistical variables affected our results. In this regard, further analysis may be needed to determine which is more important for predicting patient outcome in elderly patients, the severity of functional MR or malnutrition, and associated frailty.

It is of note that lower GNRI was significantly associated with poor primary endpoints. Several studies have reported that lower GNRI is associated with a substantially higher number of cardiac death or HF admission than higher GNRI (39, 40), of which the cut-off value is 92 (41). In this study, the cut-off value of GNRI predicting the poor outcome of the primary endpoint was 86.6, which was further lower than those previously reported. This value may reflect the higher age of the analyzed population, multiple comorbidities, and significant cognitive impairment in this study. It has been reported that malnutrition was also associated with hospitalization of HF-unrelated events including orthopedic accidents, infection, and other non-cardiac diseases. The present data were consistent with previous studies (42, 43). Additionally, the therapeutic effect of minimally invasive treatment for patients with severe AS, namely transcatheter aortic valve implantation (TAVI), was affected by the low GNRI score (44). Likewise, when minimally invasive therapy (TEER) for mitral regurgitation is considered in the future, the low GNRI score may also be a poor prognostic factor, as was the case in this study.

It is well-known that higher patients with BMI have better prognoses than those of lower patients with BMI (7). Although our result is compatible with previous findings, the coexistence of sarcopenia, frailty, and lower GNRI value underscores the importance of nutritional status in elderly patients with HF harboring moderate-to-severe MR (7, 45). Moreover, the cut-off value of BMI that predicts poor outcome was 20.3 in this study, nearly the same value as previously reported (27, 46, 47). In this regard, we may seek potential therapeutic intervention targeting body weight in elderly patients with HF harboring FMR in future studies.

Concerning multiple CR parameters, why were SPPB, 6MWT, QIS, handgrip strength test, sarcopenia indexes, and BI not effective as predictive indicators in this study? For example, the SPPB has been previously reported as a good outcome predictor in elderly patients with HF (48, 49). A relatively shorter follow-up period of this study may be associated with this difference. Moreover, we only provided a CR program during hospital admission, but not in the outpatient clinic, although we instructed every patient to perform CR at home. Further study will be needed how to provide an optimal CR program in elderly patients with HF harboring FMR.

### Limitations

This study has several limitations. First, we intended to include primary and secondary MR patients with surgically unoperated status before starting this study; however, an analysis was consequently performed only on inpatients with secondary MR. Because all the hospitalized patients with ADHF with severe primary MR, which was a tiny number, received surgical treatment, we did not enroll those patients. However, the number of surgically untreated primary patients with MR may increase in a superaged society; further study may be required. Second, this study was a prospective single-center study with relatively short-term enrollment and a small sample size. Further studies with larger samples and multicenter enrollment need to be considered. Third, this cohort was exclusively Japanese, not including other races such as African American, White, Pacific, or others.

## Conclusion

In conclusion, we found that a lower nutrition index and underweight were substantial 6-month outcome predictors in the hospitalized elderly patients with ADHF harboring with moderate-to-severe FMR.

## Data Availability Statement

The raw data supporting the conclusions of this article will be made available by the authors, without undue reservation.

## Ethics Statement

The studies involving human participants were reviewed and approved by the local institutional board at Yamaguchi Prefectural Grand Medical Center (ID: 2017-019). The patients/participants provided their written informed consent to participate in this study. Written informed consent was obtained from the individual(s) for the publication of any potentially identifiable images or data included in this article.

## Author Contributions

MM was the primary investigator for this study, collating data, and as well as the overall writing of the project. YI and TY were the project supervisor, they reviewed all documents as well as helping analyze the data, and figures and tables. KM, HN, TU, FN, and SO reviewed the manuscript and offered insights based on their own experiences. MS performed the statistical analysis. All authors gave final approval and agreed to be accountable for all aspects of work ensuring integrity and accuracy.

## Conflict of Interest

The authors declare that the research was conducted in the absence of any commercial or financial relationships that could be construed as a potential conflict of interest.

## Publisher's Note

All claims expressed in this article are solely those of the authors and do not necessarily represent those of their affiliated organizations, or those of the publisher, the editors and the reviewers. Any product that may be evaluated in this article, or claim that may be made by its manufacturer, is not guaranteed or endorsed by the publisher.

## References

[1] Singh JP Evans JC Levy D Larson MG Freed LA Fuller DL. Prevalence and clinical determinants of mitral, tricuspid, and aortic regurgitation (the Framingham Heart Study). Am J Cardiol. (1999) 83:897–902. 10.1016/S0002-9149(98)01064-9 10190406

[2] Otto CM Nishimura RA Bonow RO Carabello BA Erwin JP III Gentile F. 2020 ACC/AHA guideline for the management of patients with valvular heart disease: a report of the American College of Cardiology/American Heart Association Joint Committee on Clinical Practice Guidelines. Circulation. (2021) 143:e72–227. 10.1161/CIR.0000000000000923 33332150

[3] Stone GW Lindenfeld J Abraham WT Kar S Lim DS Mishell JM. Transcatheter mitral-valve repair in patients with heart failure. N Engl J Med. (2018) 379:2307–18. 10.1056/NEJMoa1806640 30280640

[4] Obadia JF Messika-Zeitoun D Leurent G Iung B Bonnet G Piriou N. Percutaneous repair or medical treatment for secondary mitral regurgitation. N Engl J Med. (2018) 379:2297–306. 10.1056/NEJMoa1805374 30145927

[5] Cleland JG Swedberg K Follath F Komajda M Cohen-Solal A Aguilar JC. The EuroHeart Failure survey programme– a survey on the quality of care among patients with heart failure in Europe. Part 1: patient characteristics and diagnosis. Eur Heart J. (2003) 24:442–63. 10.1016/S0195-668X(02)00823-0 12633546

[6] Shiba N Nochioka K Miura M Kohno H Shimokawa H. Trend of westernization of etiology and clinical characteristics of heart failure patients in Japan–first report from the CHART-2 study. Circ J. (2011) 75:823–33. 10.1253/circj.CJ-11-0135 21436596

[7] Anker SD Ponikowski P Varney S Chua TP Clark AL Webb-Peploe KM. Wasting as independent risk factor for mortality in chronic heart failure. Lancet. (1997) 349:1050–3. 10.1016/S0140-6736(96)07015-8 9107242

[8] Akashi YJ Springer J Anker SD. Cachexia in chronic heart failure: prognostic implications and novel therapeutic approaches. Curr Heart Fail Rep. (2005) 2:198–203. 10.1007/BF02696650 16332313

[9] Mirabel M Iung B Baron G Messika-Zeitoun D Détaint D Vanoverschelde JL. What are the characteristics of patients with severe, symptomatic, mitral regurgitation who are denied surgery? Eur Heart J. (2007) 28:1358–65. 10.1093/eurheartj/ehm001 17350971

[10] Reeves GR Whellan DJ Patel MJ O'Connor CM Duncan P Eggebeen JD. Comparison of frequency of frailty and severely impaired physical function in patients ≥60 years hospitalized with acute decompensated heart failure versus chronic stable heart failure with reduced and preserved left ventricular ejection fraction. Am J Cardiol. (2016) 117:1953–8. 10.1016/j.amjcard.2016.03.046 27156830 PMC4943325

[11] Kitzman DW Whellan DJ Duncan P Pastva AM Mentz RJ Reeves GR. Physical rehabilitation for older patients hospitalized for heart failure. N Engl J Med. (2021) 385:203–16. 10.1056/NEJMoa2026141 33999544 PMC8353658

[12] Tsutsui H Isobe M Ito H Ito H Okumura K Ono M. JCS 2017/JHFS 2017 guideline on diagnosis and treatment of acute and chronic heart failure- digest version. Circ J. (2019) 83:2084–184. 10.1253/circj.CJ-19-0342 31511439

[13] Mebazaa A Gheorghiade M Piña IL Harjola VP Hollenberg SM Follath F. Practical recommendations for prehospital and early in-hospital management of patients presenting with acute heart failure syndromes. Crit Care Med. (2008) 36:S129–39. 10.1097/01.CCM.0000296274.51933.4C 18158472

[14] Ponikowski P Voors AA Anker SD Bueno H Cleland JGF Coats AJS. 2016 ESC Guidelines for the diagnosis and treatment of acute and chronic heart failure: the Task Force for the diagnosis and treatment of acute and chronic heart failure of the European Society of Cardiology (ESC)Developed with the special contribution of the Heart Failure Association (HFA) of the ESC. Eur Heart J. (2016) 37:2129–200. 10.1093/eurheartj/ehw128 27206819

[15] Fujiwara Y Suzuki H Yasunaga M Sugiyama M Ijuin M Sakuma N. Brief screening tool for mild cognitive impairment in older Japanese: validation of the Japanese version of the Montreal Cognitive Assessment. Geriatr Gerontol Int. (2010) 10:225–32. 10.1111/j.1447-0594.2010.00585.x 20141536

[16] Bouillanne O Morineau G Dupont C Coulombel I Vincent JP Nicolis I. Geriatric Nutritional Risk Index: a new index for evaluating at-risk elderly medical patients. Am J Clin Nutr. (2005) 82:777–83. 10.1093/ajcn/82.4.777 16210706

[17] Guralnik JM Simonsick EM Ferrucci L Glynn RJ Berkman LF Blazer DG. A short physical performance battery assessing lower extremity function: association with self-reported disability and prediction of mortality and nursing home admission. J Gerontol. (1994) 49:M85–94. 10.1093/geronj/49.2.M85 8126356

[18] Kamiya K Masuda T Tanaka S Hamazaki N Matsue Y Mezzani A. Quadriceps strength as a predictor of mortality in coronary artery disease. Am J Med. (2015) 128:1212–9. 10.1016/j.amjmed.2015.06.035 26169888

[19] Guyatt GH Sullivan MJ Thompson PJ Fallen EL Pugsley SO Taylor DW. The 6-minute walk: a new measure of exercise capacity in patients with chronic heart failure. Can Med Assoc J. (1985) 132:919–23. 3978515 PMC1345899

[20] Mahoney FI Barthel DW. Functional evaluation: the barthel index. Md State Med J. (1965) 14:61–5. 10.1037/t02366-000 14258950

[21] Arai H Satake S. English translation of the Kihon Checklist. Geriatr Gerontol Int. (2015) 15:518–9. 10.1111/ggi.12397 25828791

[22] Satake S Senda K Hong YJ Miura H Endo H Sakurai T. Validity of the kihon checklist for assessing frailty status. Geriatr Gerontol Int. (2016) 16:709–15. 10.1111/ggi.12543 26171645

[23] Satake S Shimada H Yamada M Kim H Yoshida H Gondo Y. Prevalence of frailty among community-dwellers and outpatients in Japan as defined by the Japanese version of the Cardiovascular Health Study criteria. Geriatr Gerontol Int. (2017) 17:2629–34. 10.1111/ggi.13129 29265757

[24] Chen LK Liu LK Woo J Assantachai P Auyeung TW Bahyah KS. Sarcopenia in Asia: consensus report of the Asian Working Group for Sarcopenia. J Am Med Dir Assoc. (2014) 15:95–101. 10.1016/j.jamda.2013.11.025 24461239

[25] Kanda Y. Investigation of the freely available easy-to-use software 'EZR' for medical statistics. Bone Marrow Transplant. (2013) 48:452–8. 10.1038/bmt.2012.244 23208313 PMC3590441

[26] Grigioni F Enriquez-Sarano M Zehr KJ Bailey KR Tajik AJ. Ischemic mitral regurgitation: long-term outcome and prognostic implications with quantitative Doppler assessment. Circulation. (2001) 103:1759–64. 10.1161/01.CIR.103.13.1759 11282907

[27] Takiguchi M Yoshihisa A Miura S Shimizu T Nakamura Y Yamauchi H. Impact of body mass index on mortality in heart failure patients. Eur J Clin Invest. (2014) 44:1197–205. 10.1111/eci.12354 25331191

[28] Reeves GR Whellan DJ O'Connor CM Duncan P Eggebeen JD Morgan TM. A novel rehabilitation intervention for older patients with acute decompensated heart failure: the REHAB-HF pilot study. JACC Heart Fail. (2017) 5:359–66. 10.1016/j.jchf.2016.12.019 28285121 PMC5409854

[29] Tsuji K Sakata Y Nochioka K Miura M Yamauchi T Onose T. Characterization of heart failure patients with mid-range left ventricular ejection fraction-a report from the CHART-2 Study. Eur J Heart Fail. (2017) 19:1258–69. 10.1002/ejhf.807 28370829

[30] Meta-analysis Global Group in Chronic Heart Failure (MAGGIC). The survival of patients with heart failure with preserved or reduced left ventricular ejection fraction: an individual patient data meta-analysis. Eur Heart J. (2012) 33:1750–7. 10.1093/eurheartj/ehr254 21821849

[31] Koelling TM Aaronson KD Cody RJ Bach DS Armstrong WF. Prognostic significance of mitral regurgitation and tricuspid regurgitation in patients with left ventricular systolic dysfunction. Am Heart J. (2002) 144:524–9. 10.1067/mhj.2002.123575 12228791

[32] Machino-Ohtsuka T Seo Y Ishizu T Sato K Sugano A Yamamoto M. Novel mechanistic insights into atrial functional mitral regurgitation- 3-dimensional echocardiographic study. Circ J. (2016) 80:2240–8. 10.1253/circj.CJ-16-0435 27535338

[33] Kagiyama N Hayashida A Toki M Fukuda S Ohara M Hirohata A. Insufficient leaflet remodeling in patients with atrial fibrillation: association with the severity of mitral regurgitation. Circ Cardiovasc Imaging. (2017) 10:e005451. 10.1161/CIRCIMAGING.116.005451 28289019

[34] Cong T Gu J Lee AP Shang Z Sun Y Sun Q. Quantitative analysis of mitral valve morphology in atrial functional mitral regurgitation using real-time 3-dimensional echocardiography atrial functional mitral regurgitation. Cardiovasc Ultrasound. (2018) 16:13. 10.1186/s12947-018-0131-1 30126422 PMC6102822

[35] Kajimoto K Sato N Takano T. Functional mitral regurgitation at discharge and outcomes in patients hospitalized for acute decompensated heart failure with a preserved or reduced ejection fraction. Eur J Heart Fail. (2016) 18:1051–9. 10.1002/ejhf.562 27212582

[36] Kaneko H Suzuki S Uejima T Kano H Matsuno S Otsuka T. Prevalence and the long-term prognosis of functional mitral regurgitation in Japanese patients with symptomatic heart failure. Heart Vessels. (2014) 29:801–7. 10.1007/s00380-013-0448-5 24275908

[37] Wada Y Ohara T Funada A Hasegawa T Sugano Y Kanzaki H. Prognostic impact of functional mitral regurgitation in patients admitted with acute decompensated heart failure. Circ J. (2016) 80:139–47. 10.1253/circj.CJ-15-0663 26558879

[38] Davis MR Lee CS Corcoran A Gupta N Uchmanowicz I Denfeld QE. Gender differences in the prevalence of frailty in heart failure: a systematic review and meta-analysis. Int J Cardiol. (2021) 333:133–40. 10.1016/j.ijcard.2021.02.062 33657397 PMC8107129

[39] Aziz EF Javed F Pratap B Musat D Nader A Pulimi S. Malnutrition as assessed by nutritional risk index is associated with worse outcome in patients admitted with acute decompensated heart failure: an ACAP-HF data analysis. Heart Int. (2011) 6:e2. 10.4081/hi.2011.e2 21977302 PMC3184716

[40] Narumi T Arimoto T Funayama A Kadowaki S Otaki Y Nishiyama S. Prognostic importance of objective nutritional indexes in patients with chronic heart failure. J Cardiol. (2013) 62:307–13. 10.1016/j.jjcc.2013.05.007 23806549

[41] Kinugasa Y Kato M Sugihara S Hirai M Yamada K Yanagihara K. Geriatric nutritional risk index predicts functional dependency and mortality in patients with heart failure with preserved ejection fraction. Circ J. (2013) 77:705–11. 10.1253/circj.CJ-12-1091 23182759

[42] Beaudart C Reginster JY Petermans J Gillain S Quabron A Locquet M. Quality of life and physical components linked to sarcopenia: the SarcoPhAge study. Exp Gerontol. (2015) 69:103–10. 10.1016/j.exger.2015.05.003 25979160

[43] Cheng MH Chang SF. Frailty as a risk factor for falls among community dwelling people: evidence from a meta-analysis. J Nurs Scholarsh. (2017) 49:529–36. 10.1111/jnu.12322 28755453

[44] Koseki K Yoon SH Kaewkes D Koren O Patel V Kim I. Impact of the geriatric nutritional risk index in patients undergoing transcatheter aortic valve implantation. Am J Cardiol. (2021) 157:71–8. 10.1016/j.amjcard.2021.07.016 34373077

[45] Khan H Kalogeropoulos AP Georgiopoulou VV Newman AB Harris TB Rodondi N. Frailty and risk for heart failure in older adults: the health, aging, and body composition study. Am Heart J. (2013) 166:887–94. 10.1016/j.ahj.2013.07.032 24176445 PMC3844525

[46] Hamaguchi S Tsuchihashi-Makaya M Kinugawa S Goto D Yokota T Goto K. Body mass index is an independent predictor of long-term outcomes in patients hospitalized with heart failure in Japan. Circ J. (2010) 74:2605–11. 10.1253/circj.CJ-10-0599 21060207

[47] Matsushita M Shirakabe A Hata N Shinada T Kobayashi N Tomita K. Association between the body mass index and the clinical findings in patients with acute heart failure: evaluation of the obesity paradox in patients with severely decompensated acute heart failure. Heart Vessels. (2017) 32:600–8. 10.1007/s00380-016-0908-9 27778068

[48] Guralnik JM Ferrucci L Pieper CF Leveille SG Markides KS Ostir GV. Lower extremity function and subsequent disability: consistency across studies, predictive models, and value of gait speed alone compared with the short physical performance battery. J Gerontol A Biol Sci Med Sci. (2000) 55:M221–31. 10.1093/gerona/55.4.M221 10811152 PMC12149745

[49] Chiarantini D Volpato S Sioulis F Bartalucci F Del Bianco L Mangani I. Lower extremity performance measures predict long-term prognosis in older patients hospitalized for heart failure. J Card Fail. (2010) 16:390–5. 10.1016/j.cardfail.2010.01.004 20447574

