# Corrigendum: Malnutrition and Frailty Are Critical Determinants of 6-Month Outcome in Hospitalized Elderly Patients With Heart Failure Harboring Surgically Untreated Functional Mitral Regurgitation

**DOI:** 10.3389/fcvm.2022.856009

**Published:** 2022-02-17

**Authors:** Masakazu Miura, Shinichi Okuda, Kazuhiro Murata, Hitoshi Nagai, Takeshi Ueyama, Fumiaki Nakao, Mototsugu Shimokawa, Takeshi Yamamoto, Yasuhiro Ikeda

**Affiliations:** ^1^Department of Rehabilitation, Yamaguchi Prefectural Grand Medical Center, Hofu, Japan; ^2^Division of Nursing and Laboratory Science, Yamaguchi University Graduate School of Medicine, Ube, Japan; ^3^Ultrasonography Center, Yamaguchi Prefectural Grand Medical Center, Hofu, Japan; ^4^Department of Cardiology, Yamaguchi Prefectural Grand Medical Center, Hofu, Japan; ^5^Department of Biostatistics, Yamaguchi, University Graduate School of Medicine, Ube, Japan

**Keywords:** functional mitral regurgitation (FMR), heart failure, older people, body mass index, malnutrition, frailty

In the published article, there was an error regarding the affiliation for **Mototsugu Shimokawa**. As well as having affiliation(s) **2**, they should also have **Department of Biostatistics, Yamaguchi, University Graduate School of Medicine, Ube, Japan**.

The authors apologize for this error and state that this does not change the scientific conclusions of the article in any way. The original article has been updated.

## Publisher's Note

All claims expressed in this article are solely those of the authors and do not necessarily represent those of their affiliated organizations, or those of the publisher, the editors and the reviewers. Any product that may be evaluated in this article, or claim that may be made by its manufacturer, is not guaranteed or endorsed by the publisher.

