# Supplementary material for: Malnutrition and Frailty Are Critical Determinants of 6-Month Outcome in Hospitalized Elderly Patients With Heart Failure Harboring Surgically Untreated Functional Mitral Regurgitation
Source: Front Cardiovasc Med. 2021 Dec 2;8:764528. doi: 10.3389/fcvm.2021.764528 (PMC8674489; doi:10.3389/fcvm.2021.764528)
Supplement: Supplementary file 1 [file Data_Sheet_1.PDF]

Table S1 English translation of the Kihon Checklist

No.	Questions	Answer	
1	Do you go out by bus or train by yourself?	☐ 0 Yes	☐ 1 No
2	Do you go shopping to buy daily necessities by yourself?	☐ 0 Yes	☐ 1 No
3	Do you manage your own deposits and savings at the bank?	☐ 0 Yes	☐ 1 No
4	Do you sometimes visit your friends?	☐ 0 Yes	☐ 1 No
5	Do you turn to your family or friends for advice?	☐ 0 Yes	☐ 1 No
6	Do you normally climb stairs without using handrail or wall for support?	☐ 0 Yes	☐ 1 No
7	Do you normally stand up from a chair without any aids?	☐ 0 Yes	☐ 1 No
8	Do you normally walk continuously for 15 minutes?	☐ 0 Yes	☐ 1 No
9	Have you experienced a fall in the past year?	☐ 1 Yes	☐ 0 No
10	Do you have a fear of falling while walking?	☐ 1 Yes	☐ 0 No
11	Have you lost 2 kg or more in the past 6 months?	☐ 1 Yes	☐ 0 No
12	Height: cm, weight: kg, BMI: kg/m^2 If BMI is less than 18.5, this item is scored.	☐ 1 Yes	☐ 0 No
13	Do you have any difficulties eating tough foods compared to 6 months ago?	☐ 1 Yes	☐ 0 No
14	Have you choked on your tea or soup recently?	☐ 1 Yes	☐ 0 No
15	Do you often experience having a dry mouth?	☐ 1 Yes	☐ 0 No
16	Do you go out at least once a week?	☐ 0 Yes	☐ 1 No
17	Do you go out less frequently compared to last year?	☐ 1 Yes	☐ 0 No
18	Do your family or your friends point out your memory loss? e.g."You ask the same question over and over again."	☐ 1 Yes	☐ 0 No
19	Do you make a call by looking up phone numbers?	☐ 0 Yes	☐ 1 No
20	Do you find yourself not knowing today's date?	☐ 1 Yes	☐ 0 No
21	In the last 2 weeks have you felt a lack of fulfilment in your daily life?	☐ 1 Yes	☐ 0 No
22	In the last 2 weeks have you felt a lack of joy when doing the things you used to enjoy?	☐ 1 Yes	☐ 0 No
23	In the last 2 weeks have you felt difficulty in doing what you could do easily before?	☐ 1 Yes	☐ 0 No
24	In the last 2 weeks have you felt helpless?	☐ 1 Yes	☐ 0 No
25	In the last 2 weeks have you felt tired without a reason?	☐ 1 Yes	☐ 0 No

Working Group on Frailty in Japanese Geriatrics Society. BMI, body mass index.

Reference : Arai H, Satake S. English translation of the Kihon Checklist. Geriatrics & gerontology international. 2015;15(4):518-9.
