# Supplementary material for: Malnutrition and Frailty Are Critical Determinants of 6-Month Outcome in Hospitalized Elderly Patients With Heart Failure Harboring Surgically Untreated Functional Mitral Regurgitation
Source: Front Cardiovasc Med. 2021 Dec 2;8:764528. doi: 10.3389/fcvm.2021.764528 (PMC8674489; doi:10.3389/fcvm.2021.764528)
Supplement: Supplementary file 2 [file Data_Sheet_2.PDF]

Table S2 Univariate and Multivariate Cox Proportional Hazard Analyses to Predict Composite Endpoint After Discharge of ADHF patients with FMR

Variables	Univariate			Multivariate		
	HR	95%CI	p-value	HR	95%CI	p-value
Age	1.489	0.862 - 2.572	0.154	1.058	1.006 – 1.112	0.027
Male sex	1.149	0.670 - 1.969	0.614			
BMI	0.430	0.243 - 0.760	0.004			
Living alone	1.271	0.690 - 2.341	0.442			
Nursing care insurance	1.055	0.611 - 1.824	0.847			
Length of hospital stay	1.443	0.837 - 2.489	0.187			
Readmission	1.467	0.852 - 2.526	0.167			
SBP	0.990	0.976 - 1.005	0.206			
Orthopedic disease	1.007	0.574 - 1.766	0.980			
Stroke	1.237	0.622 - 2.462	0.545			
CKD	0.975	0.512 - 1.856	0.938			
Hypertension	0.911	0.531 - 1.563	0.735			
DM	0.946	0.520 - 1.719	0.854			
Atrial fibrillation	0.849	0.487 - 1.480	0.564			
IHD	1.524	0.892 - 2.603	0.123			
Return to home	0.847	0.453 - 1.585	0.604			
NYHA IV	0.840	0.479 - 1.474	0.544			
Grade 0 MR (reference)						
Grade I MR	0.550	0.180 - 1.684	0.295			
Grade II MR	0.635	0.242 - 1.666	0.356			
Grade III MR	0.644	0.233 - 1.782	0.397			
Grade IV MR	1.156	0.135 - 9.903	0.895			
Grade III MR or greater	1.117	0.633 - 1.972	0.702			
LVEF	0.582	0.335 - 1.010	0.054	0.971	0.945 - 0.997	0.030
LVDd	1.023	0.993 - 1.054	0.136			
LVDs	1.018	0.992 - 1.044	0.174			
LAD	1.444	0.837 - 2.492	0.187			
LAVI	1.249	0.711 - 2.192	0.439			
E/e' ratio	1.064	0.585 - 1.935	0.839			
E/A ratio	0.710	0.365 - 1.382	0.314			
TRPG	0.911	0.522 - 1.592	0.744			
Tethering height	1.062	0.953 - 1.184	0.276			
Vena Contracta	1.130	0.932 - 1.371	0.214			
EROA	2.131	1.121 - 4.051	0.021			

TAPSE	1.389	0.673 - 2.868	0.374			
Serum albumin	0.854	0.496 - 1.471	0.570			
Serum creatinine	0.993	0.579 - 1.703	0.980			
eGFR	0.740	0.429 - 1.278	0.281			
Serum hemoglobin	0.761	0.442 - 1.312	0.326			
BNP	1.881	1.075 - 3.291	0.027			
GNRI	0.511	0.292 - 0.895	0.019	0.932	0.887 - 0.979	0.005
ACE-I/ARB	0.673	0.386 - 1.174	0.163			
β-blocker	1.024	0.569 - 1.843	0.937			
Loop diuretics	1.045	0.549 - 1.990	0.892			
MRAs	1.324	0.763 - 2.296	0.319			
Tolvaptan	2.334	1.359 - 4.007	0.002			
SPPB	0.740	0.424 - 1.291	0.289			
Handgrip strength	0.938	0.547 - 1.609	0.815			
QIS	1.281	0.739 - 2.220	0.377			
Sarcopenia	1.806	0.904 - 3.608	0.094			
BI	0.840	0.487 - 1.449	0.530			
MoCA-J	0.849	0.475 - 1.516	0.579			
KCL	1.482	0.854 - 2.571	0.162			
6MWT	0.774	0.447 - 1.337	0.358			

The multivariate Cox proportional hazard analysis results were shown with adjustment of the selected covariates, including variables with a p-value of less than 0.20 in univariate analysis, age, sex, BMI, SBP, LVEF, IHD, EROA, grade III MR or greater, and SPPB. Abbreviations indicate HR: hazard ratio; CI: confidence interval; ADHF: acute decompensated heart failure; FMR: functional mitral regurgitation; MR: mitral regurgitation; BMI: body mass index; SBP: systolic blood pressure; NYHA: New York Heart Association; CKD: chronic kidney disease; DM: diabetes mellitus; IHD: ischemic heart disease; LVEF: left ventricular ejection fraction; LVDd: left ventricular end-diastolic dimension; LVDs: left ventricular end-systolic dimension; LAD: left atrial dimension; LAVI: left atrial volume index; TRPG: transtricuspid pressure gradient; TAPSE: tricuspid annular plane systolic excursion; EROA: effective regurgitant orifice area; eGFR: estimated glomerular filtration rate; BNP: brain natriuretic peptide; GNRI: geriatric nutrition risk index; ACE-I/ARB: angiotensin-converting enzyme-inhibitor/angiotensin II receptor blocker; MRAs: mineralocorticoid receptor antagonists; SPPB: short physical performance battery; QIS: quadriceps isometric strength; BI: Barthel Index; MoCA-J: The Japanese version of Montreal cognitive assessment; KCL: Kihon Checklist (See Supplement File #1); 6MWT: six minutes walking test.
