# Supplementary material for: Malnutrition and Frailty Are Critical Determinants of 6-Month Outcome in Hospitalized Elderly Patients With Heart Failure Harboring Surgically Untreated Functional Mitral Regurgitation
Source: Front Cardiovasc Med. 2021 Dec 2;8:764528. doi: 10.3389/fcvm.2021.764528 (PMC8674489; doi:10.3389/fcvm.2021.764528)
Supplement: Supplementary file 3 [file Data_Sheet_3.PDF]

Table S3 Univariate and Multivariate Cox Proportional Hazard Analyses to Predict HF-related Endpoint After Discharge of ADHF patients with FMR

Variables	Univariate			Multivariate		
	HR	95%CI	p-value	HR	95%CI	p-value
Age	1.590	0.765 - 3.304	0.214			
Male sex	0.784	0.378 - 1.628	0.514			
BMI	0.487	0.231 - 1.027	0.059	0.739	0.614 -0.890	0.001
Living alone	0.876	0.358 - 2.144	0.772			
Nursing care insurance	1.144	0.555 - 2.358	0.715			
Length of hospital stay	1.212	0.548 - 2.296	0.754			
Readmission	2.261	1.058 - 4.832	0.035			
SBP	1.001	0.982 - 1.021	0.893			
Orthopedic disease	1.209	0.582 - 2.510	0.611			
Stroke	1.329	0.543 - 3.253	0.533			
CKD	1.462	0.669 - 3.194	0.341			
Hypertension	0.918	0.448 - 1.881	0.815			
DM	0.876	0.390 - 1.968	0.749			
Atrial fibrillation	1.234	0.602 - 2.530	0.566			
IHD	1.797	0.897 - 3.599	0.098	2.732	1.056 - 7.067	0.038
Return to home	0.902	0.387 - 2.103	0.811			
NYHA IV	0.607	0.295 - 1.249	0.175			
Grade 0 MR (reference)						
Grade I MR	0.526	0.088 - 3.150	0.482			
Grade II MR	0.872	0.197 - 3.867	0.857			
Grade III MR	1.240	0.274 - 5.606	0.397			
Grade IV MR	2.798	0.253 - 30.900	0.401			
Grade III MR or greater	1.748	0.869 - 3.517	0.117			
LVEF	0.600	0.289 - 1.248	0.172			
LVDd	0.999	0.960 - 1.039	0.963			
LVDs	1.006	0.973 - 1.040	0.719			
LAD	1.529	0.735 - 3.182	0.256			
LAVI	1.710	0.800 - 3.656	0.167			
E/e' ratio	1.326	0.581 - 3.026	0.502			
E/A ratio	1.299	0.302 - 5.578	0.725			
TRPG	1.252	0.601 - 2.607	0.548			
Tethering height	1.091	0.947 - 1.258	0.228			
Vena Contracta	1.143	0.901 - 1.451	0.270			
EROA	1.275	0.532 - 3.058	0.586			

TAPSE	1.772	0.697 - 4.504	0.230
Serum Albumin	0.620	0.295 - 1.303	0.207
Serum Creatinine	1.116	0.544 - 2.286	0.765
eGFR	0.518	0.242 - 1.106	0.089
Serum hemoglobin	0.569	0.270 - 1.198	0.138
BNP	2.336	1.093 - 4.994	0.029
GNRI	0.432	0.201 - 0.926	0.031
ACE-I /ARB	0.802	0.375 - 1.715	0.570
β-blocker	1.029	0.471 - 2.251	0.942
Loop diuretics	1.518	0.581 - 3.969	0.394
MRAs	0.997	0.466 - 2.131	0.994
Tolvaptan	2.965	1.445 - 6.083	0.003
SPPB	0.515	0.236 - 1.125	0.096
Handgrip strength	0.600	0.285 - 1.260	0.177
QIS	0.923	0.446 - 1.913	0.830
Sarcopenia	2.291	0.844 - 6.217	0.103
BI	0.941	0.459 - 1.928	0.867
MoCA-J	0.794	0.371 - 1.698	0.552
KCL	2.647	1.238 - 5.658	0.012
6MWT	0.600	0.283 - 1.270	0.182

The multivariate Cox proportional hazard analysis results were shown with adjustment of the selected covariates, including variables with a p-value of less than 0.20 in univariate analysis, age, sex, BMI, SBP, LVEF, IHD, EROA, grade III MR or greater, and SPPB.
